# Predictive Effect of *Helicobacter pylori* in Gastric Carcinoma Development: Systematic Review and Quantitative Evidence Synthesis

**DOI:** 10.3390/medicines8010001

**Published:** 2021-01-05

**Authors:** Laurens Holmes, Jasmine Rios, Betyna Berice, Jacqueline Benson, Nastocia Bafford, Kadedrah Parson, Daniel Halloran

**Affiliations:** 1 Nemours Healthcare System for Children, Wilmington, DE 19803, USA; jasmine.rios@yale.edu (J.R.); tynaberice@gmail.com (B.B.); jabenson@pennmedicine.upenn.edu (J.B.); nastocia.bafford@students.jsums.edu (N.B.); kadedrahparson@gmail.com (K.P.); dhallor@udel.edu (D.H.); 2 Department of Biological Sciences, University of Delaware, Newark, DE 19716, USA; 3 History of Science and Medicine Department, Yale University, New Haven, CT 06511, USA; 4 Master of Public Health, Dr. Kiran C. Patel College of Osteopathic Medicine, Nova Southeastern University, Davie, FL 33328, USA; 5 Master of Public Health Program, Perelman School of Medicine, University of Pennsylvania, Philadelphia, PA 19104, USA

**Keywords:** *Helicobacter pylori* (*H. pylori*), gastric carcinoma, gastritis, gastric ulceration, QES

## Abstract

*Helicobacter pylori* (*H. pylori*) is a bacterial pathogen implicated in gastritis, gastric ulceration, and gastric carcinoma. This study aimed to synthesize literature in providing evidence on the causative role of *H. pylori* in gastric carcinoma development. This study is based on assessing public literature using an applied meta-analysis, namely, quantitative evidence synthesis (QES). The analytic procedure uses DerSimonian-Laird, including assessing heterogeneity. The QES also utilizes meta-regression and the environmental effect associated with *H. pylori* in gastric cancer development. Eighteen studies are included in the QES. There is increased prevalence of *H. pylori* exposure among the cases. The heterogeneity between the CES and individual effect sizes is also significant. Despite controlling for the confoundings, there is increased exposure to *H. pylori* among the gastric cancer cases, regardless of the differences in the geographic location. *H. pylori* in this synthesized literature illustrates the contributory role of this microbe in gastric carcinoma. Additionally, regardless of geographic locale, namely, South Korea or Spain, *H. pylori* is implicated in gastric cancer development.

## 1. Introduction

*Helicobacter pylori* (*H. pylori*) is a bacterial pathogen associated with the gastrointestinal (GI) tract of over 50% of the world’s population [1]. *H. pylori* infection typically occurs in early childhood and can often present with no symptoms [2]. In symptomatic cases, nausea, vomiting, abdominal pain, and peptic ulcers are among the most common clinical manifestations [2]. Furthermore, *H. pylori* is recognized as a type I carcinogen, and the most common cause of bacterial-induced malignancies [3]. *H. pylori* colonization in the GI tract is a well-established risk factor for malignancies, specifically of the stomach [4]. *H. pylori* has been illustrated to colonize cells in the GI tract, due to its motility with flagella and its ability to utilize gastric acids for survival [5]. Epidemiologic and clinical data have extensively attempted to delineate the contributory risk of *H. pylori* in gastric adenocarcinoma, demonstrating its role in up to 75% of non-cardia gastric malignancies and up to 98% of gastric cardia malignancies [4]. While the incidence of gastric malignancy has decreased over time, it remains the third most common cause of cancer-related deaths worldwide. Understanding the correlation between *H. pylori* infection during childhood and gastric adenocarcinoma in late adulthood needs to be elucidated for the potential of primary clinical intervention, treatment, and prevention.

Although *H. pylori* infection early in life is a strong risk factor for gastric cancer, an estimated 2% of those infected with *H. pylori* worldwide developed gastric malignancy [6]. Although a variance in studies of the efficiency of *H. pylori* eradication or screening efforts towards reducing gastric cancer risk has been observed, most studies on efficiency suggest that children with family histories of gastric cancer benefit from *H. pylori* eradication or screening efforts; however, little is known of other groups that could benefit from early intervention [7]. The complexities in this research area arise primarily because of the extensive and challenging processes proposed from infection to cancer and the genetic diversity of *H. pylori* worldwide. Further research is needed to explain subpopulation variance in the risk of malignancy from *H. pylori* infection more robustly.

### 1.1. H. pylori Infection and Gastric Cancer

*H. pylori* primarily infects the epithelial cells in the lining of the stomach and can survive in humans for decades by inhibiting the immune system responsiveness and inducing chronic inflammatory responses [8]. Over the past three decades, inflammation was thought to cause DNA damage leading to peptic ulcer disease, ultimately causing the turnover of cells, predisposing them to malignant neoplasm [9]. The outer inflammatory protein A (oipA), which is a virulence factor in *H. pylori*, has been observed in chronic gastritis and gastric carcinoma. In addition to oipA, the vacuolating cytotoxin A (vacA) has been observed as a virulence factor in *H. pylori*. VacA has been associated with apoptosis in epithelial cells of the gastric mucosa, immune system responsiveness, as well as the modulation of the permeability of polarized epithelial cells monolayers [10,11,12,13]. Historically, *H. pylori* has been implicated in gastritis, which reflects the inflammation of the gastric intestinal mucosa. Given endotoxin elaboration and other inflammatory exudates, the colonization of the gastric mucosa by *H. pylori* has been observed with gastric atrophy [14,15]. The chronic *H. pylori* involvement in the gastric intestinal tract, namely, the stomach, has a tendency to induce DNA damage that may result in intestinal metaplasia [16,17]. With *H. pylori* involvement in the gastric intestinal pH alteration, dysplasia has been observed in patients with *H. pylori* infection [18,19]. This integrative model also helps explain the interest in *H. pylori*’s potential role in carcinogenesis of non-gastric malignancies. Furthermore, studies have shown its associated risk with non-Hodgkin’s lymphoma, pancreatic cancer, colorectal cancer, and even lung cancer with varied responses [20,21,22,23].

Conversely, to the inflammatory model, more recent findings demonstrate that several tumor suppressor genes are consistently downregulated among children diagnosed with *H. pylori* [24,25]. This finding suggests there may be other potential pathways in which *H. pylori* infection can cause malignancy, emphasizing the greater motivation for continued follow-up, screenings, and/or treatment of infected children. This is particularly important when considering the varied relationship between *H. pylori* and cancer worldwide.

To date, epidemiological studies vary on the explicit pathway of *H. pylori* infection to gastric carcinoma. Among the worldwide population residing in low- or middle-income countries, up to 80% are infected with *H. pylori* [1]. Conversely, high-income countries have observed a steady decline in *H. pylori* infection [26]. Disparities in *H. pylori* infection are explained by the increased risk of poor living conditions (i.e., crowded living conditions, scarce sanitation facilities, lower-middle SES) [27]. However, the incidence of gastric cancer and other GI malignancies do not follow these same patterns and predictor variables, implying multfactoriality in gastric cancer carcinogenesis. Notable differences between *H. pylori* and gastric cancer prevalence can be observed more starkly between subpopulations in low-income countries [28]. In these countries, the prevalence of *H. pylori* infection is high nationally, and yet they report regionally-varied rates of gastric cancer [28]. These incongruencies represent the intersection of several cancer-modulating risk factors: Genetic variation of *H. pylori*, co-infection, and dietary patterns [28].

### 1.2. Subpopulation Variance of H. pylori and Gastric Cancer

Existing literature on cancer-modulating risk factors attempt to elucidate differences between different regional groups; however, few have attempted a review to quantitatively assess the validity of such differences. Current research offers a variety of potential mediating factors that may influence the observed variance in gastric cancer risk given *H. pylori* infection.

Globally, regional risk factors like diet or co-morbidities are thought to modulate the progression of *H. pylori* infection to malignancy. Diets higher in antioxidant micronutrients and increased risk of helminth infection have been observed in southern India to be protective against *H. pylori*-mediated gastric cancer [29]. These two factors alter the GI microbiome and immune response in a way that buffers against chronic gastritis [28,29]. Additionally, in China, preference for spicy, salty, and high -temperature foods increased the risk of gastric cancer [30].

Research on economic and demographic factors influencing gastric cancer risk has not been very well-established. Socioeconomic status alone is not a clear causal risk factor to fully explain differences in *H. pylori* infections that may lead to gastric cancer between countries. Additionally, the risk of developing gastric cancer due to *H. pylori* infection does not appear to follow socioeconomic trends in the United States (US), whereas race and region may be greater influences [31,32]. Differences in *H. pylori* infection and gastric cancer between race/ethnicity have been observed in a few studies. In the US, Black/African American and Native American patients had the highest risk of being diagnosed with gastric cancer post-*H. pylori* infection [33,34]. Another study performed in Malaysia observed that East Asian ethnicity was associated with a higher risk of gastric cancer than the native Malaysian and Indian populations [35]. Racial and ethnic disparities in gastric cancer risk may be due to other social factors not identified in present studies including racism, healthcare infrastructure, or housing. Currently, racial//ethnic differences in gastric cancer risk may be best explained by the genetic diversity of *H. pylori*.

Genomic research has revealed extensive genetic diversity in *H. pylori* strains. This research has shown regional subpopulation variants of *H. pylori* that co-evolved with different ethnic groups in various regions of the world [36]. Previous studies suggest that regional strains variably predispose populations to gastric adenocarcinoma, explaining mosaic prevalence in countries with varied ancestry, like Colombia [36]. Furthermore, due to *H. pylori* genetic variance, it is often useful to classify the bacteria into cagA positive or negative strains. A study based in the US suggested that a higher prevalence of cagA positive strains of *H. pylori* are greater among those of African ancestry [31]. cagA positive strains induce cell migration and are associated with more adverse health outcomes, potentially explaining racial patterns in gastric cancer post-*H. pylori* infection [3].

Currently, there are many gaps in the scientific understanding of the association between pediatric *H. pylori* infection and adult malignancies, especially regarding subpopulations that may be predisposed or more susceptible to developing such malignancies, like gastric cancer. This QES aimed to observe the association between *H. pylori* and the development of gastric cancer in two culturally distinct regions in an attempt to fill this gap. We selected South Korea and Spain to represent East Asia and Western Europe, respectively. These countries were chosen given their comparability in *H. pylori* seroprevalence, population size, development index, life expectancy, and national income level. To our knowledge, there have been few studies examining the geographic implications of the correlation between *H. pylori* and gastric cancer, and none directly comparing two countries. The primary aim of this study was to provide evidence to support or refute the current claim that *H. pylori* infection increases the risk of gastric carcinoma development. The secondary aim was to identify geographic variance by comparing the risk of gastric cancer given prior *H. pylori* infection in two culturally and geographically distinct countries.

## 2. Materials and Methods

### 2.1. Study Design

Evidence-based literature findings are dependent on the ability to synthesize published literature for evidence discovery, scientific opinion, and scientific statement. A classic example of evidence-based data involves the application of meta-analysis in such discovery. However, the limitations of traditional meta-analysis motivated the development of quantitative evidence synthesis (QES), as an applied meta-analysis. The utilization of QES provides a common effect size (CES) to provide a summary estimate on the carcinogenic effect of the *H. pylori* bacterium on gastric cells, thus allowing for the scientific statement of the epigenomic variance of *H. pylori*-induced gastric carcinoma in Spain and South Korea [37,38,39].

### 2.2. Design Rationale

The objectives of QES are to (1) minimize the occurrence of random errors and to (2) marginalize measurement errors, which largely influence point estimates by moving away or toward the null. As studies have measurement errors, a QES analyzes these differences between studies and accounts for the errors. Furthermore, studies in medicine and public health are often conducted with small samples, and may have increased random error occurrence. The QES is a method of summarizing studies, increases the study or sample size from individual studies, and therefore, minimizes random error and increases the generalizability of the findings from multiple studies. Furthermore, QES integrates results to identify patterns, and to some extent, establishes causation, to imply a reliable and causal inference. In effect, the utilization of QES generates scientific data that is accumulative and reliable to public health and medicine [38,39].

### 2.3. QES Significance and Relevance

The methodology used in a QES varies from traditional meta-analyses. While meta-analysis utilizes fixed and random effect methods, a QES uses a random effect method and examines heterogeneity after the pool estimates. The fixed effect method is only applicable to QES when the combined studies or publications are from multicenter trials where the study protocols are identical. However, when utilizing studies from various backgrounds, such as *H. pylori* and gastric carcinoma in Spain and South Korea, the introduction of observation and measurement errors may be observed, thus limiting such combination without accounting for variability between studies. Here, the random effect method is used to minimize the between studies variability, making it unique QES applications. Furthermore, scientific endeavor accumulates literature in medicine and public health and analyzes results provided by the confounding and contradicting results. QES uses these integrations for public health and clinical decision-making.

### 2.4. QES Dynamism and Intervention Mapping

A specific facet of QES is temporality, indicating that the findings portrayed by QES accumulate and build over time. For example, if a QES was performed on the implication of *H. pylori* as an exposure function of gastric carcinoma, this study must continue to add findings, account for the time of conduct, and reanalyze the data for contrasting or negative findings. Furthermore, the introduction of data on epigenomic modulation (i.e., physical, in utero, social, endocrine, neurobiological, or environmental) informs a QES by changing the direction of the previously collected data, suggestive of the change in the results of QES.

Scientific discovery is dynamic (continually being altered and modified as new evidence accumulates), indicating the emergence of new data, causing a shift in the evidence. The scientific community must not wait until evidence accumulates to a point in which further additions are required in relation to evidence discovery to initiate an intervention. Consequently, QES can inform and generate the knowledge required in risk identification (specific risk prediction), subclinical and clinical disease progression, disease prognosis, and control and disease prevention at the population level. The effectiveness of QES in evidence-based practice is dependent on its ability to address treatment effects, preventions, or controls of a given condition and is dependent on the baseline data, which continues to change as new data emerge.

### 2.5. Search Engine and Strategies

The current QES involves assessing published literature on the exposure function of *H. pylori* in gastric carcinoma development between the years 1997–2018. The online database search was conducted in June, July, and August 2020. Initial data recovery was performed with MEDLINE via PubMed. Supplemental data recovery was performed using Embase via OVID, removing duplicates as the search occurred. Search terms were created using medical subject headings (MeSH) and terms used in previously available literature reviews of *H. pylori* as a carcinogen to maximize sensitivity: (stomach cancer OR stomach neoplasm OR gastric cancer OR gastric carcinoma OR gastric neoplasm OR cancer of stomach) AND (*helicobacter pylori* OR *h pylori* OR *pylori* infection OR *campylobacter pylori*) AND ((1) (South Korea OR Korea OR Korean) OR (2) (Spain OR Spanish).

Additionally, hand searches were performed through reference lists of relevant articles. This process involved the identification of papers, and where necessary, contact with the authors via email communication for further clarification of the findings.

### 2.6. Study Eligibility

Abstracts were screened for utilization in the full-text review. Eligible articles met the following inclusion criteria: (1) Study published in English up until June 2019; (2) study investigates risk factors for gastric cancer including *H. pylori* infection status; (3) study investigates early or late state gastric neoplasm subjects, rather than precancerous lesions which do not always progress into gastric cancer; (4) study subjects were not exposed to *H. pylori* eradication efforts as a part of the experiment; and (5) study included quantitative data, including parameter values (odds ratio, risk ratio, relative risk).

**Figure 1:**
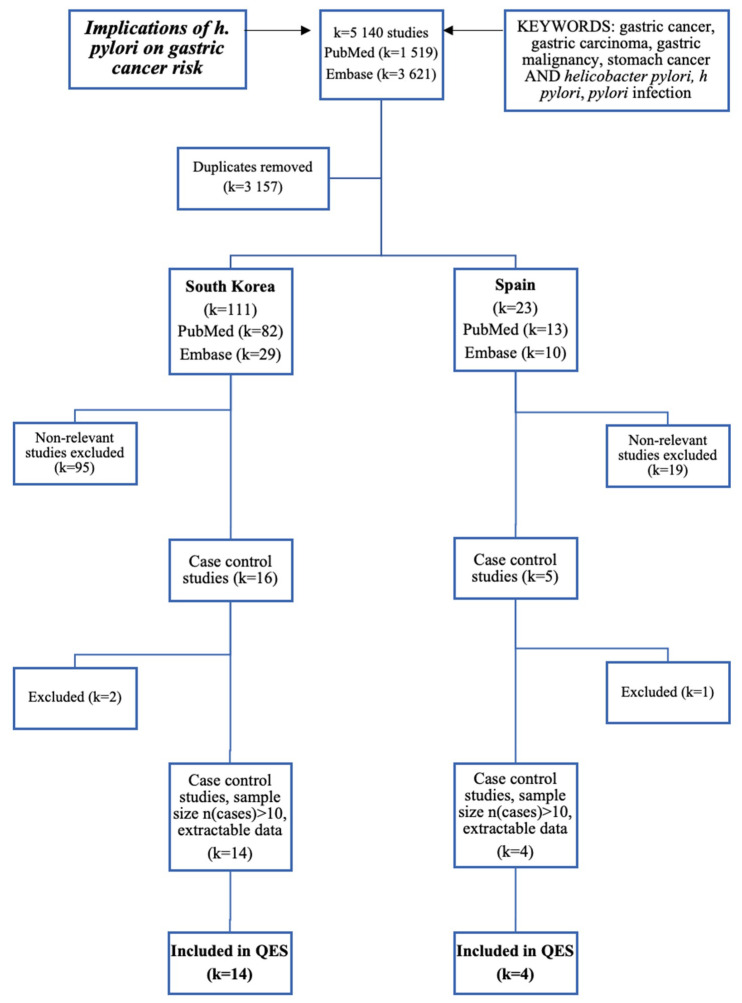
A schematic representing the inclusion or exclusion of studies from the QES. All studies were published between 1997–2018 and were located using the described search terms. In total, 14 studies from South Korea were included, and four studies from Spain were included.

Eligible studies emerged primarily as case-control studies, due to the high prevalence of *H. pylori* infection (exposure) compared to the prevalence of gastric carcinoma (outcome). Furthermore, additional exclusion criteria involved studies with <10 controls, a case-control ratio of <1:1, and controls at least based on age, sex, and region. If the study alluded to the existence of quantitative data, but was not available in the text, the authors were contacted. Detailed information about study eligibility is illustrated in Figure 1.

### 2.7. Data Extraction

The information from this QES was extracted by three authors independently, namely, DH, BB, and JR. The variables extracted were based on the quantification of the independent variable, outcome variable, and the measures of precision, namely, 95% and/or 99% confidence interval, as well as the random error quantification value, namely, the probability value (*p*-value). The inter-rater agreement between DH and BB was based on the kappa-statistic and was obtained; κ = 1.00, implying high reliability concerning concordance.

### 2.8. Study Variables

The independent variable as the main predictor of gastric carcinoma in this QES was *H. pylori*. As *H. pylori* has a role in gastritis, the transformation of the pre-oncogene to the oncogene, and inactivation of the tumor suppressor gene, leading subsequent inhibition of apoptosis, this QES focuses mainly on this pathogen, although other risk factors such as barbeque (BBQ) foods, processed foods, family history, and smoking can lead to gastric cancer. The *H. pylori* variable was measured on a discrete scale, implying the absence or presence of infection, which allowed for the regression model in the individual studies that constituted the QES. Another variable examined was the geographical locale, namely, Spain (Western Europe) and South Korea (Eastern Asia).

### 2.9. Data Analysis

Before the descriptive and inferential statistics, the individual studies that comprised the QES were examined for data tabulation and the relevance of the effect size for the design. The descriptive statistic was performed using frequency and percentages.

The QES involves hypotheses testing of the hypotheses from the individual studies, implying an inferential statistic that allows for the generalizability of the findings. To test the null hypothesis of no association between *H. pylori* and gastric carcinoma, the random effect meta-analytic procedure of Dersimonian-Laird was used [40,41]. This analytic procedure allows for the adjustment between the CES and the individual effect size variations based on the weight of the studies. In addition, the heterogeneity test was performed after the CES pool estimate. This test examines the variation between the CES and the individual effect sizes using a chi-squared (χ^2^)-statistic and the degrees of freedom. Furthermore, the null hypothesis that the CES equals zero was examined using the z-statistic. This test statistic determines whether the observed CES was zero, implying no association between *H. pylori* and gastric carcinoma. The null hypothesis was rejected, as the z-statistic and the random error quantification was high and less than 0.05, respectively.

To graphically illustrate these findings, we utilize a forest plot, which characterized the individual studies, the effect sizes, and the 95% confidence interval (CI). While the individual effect sizes were presented by dots and the Cis were displayed as lines, the CES represented the diamond in the forest plot. All test statistics were two-tailed. The type-1 error tolerance was set at 5% (95% CI). The entire analysis was performed using Stata v16 (Stata Corporation, College Station, TX, USA).

Three authors (J.R., D.H., and B.B.) determined the eligible studies’ inclusion in the QES by observing study designs, sampling procedures, clarity and specificity of objectives, and appropriate statistical analyses. Furthermore, studies were assessed for confounding factors that may have influenced the outcomes, along with any potential bias, including selection, information, and misclassification biases. This technique was adequately comparable to the preferred method of reporting for systematic reviews (PRISMA statement) [39].

## 3. Results

Evidence-based synthesis remains a pathway to understanding a disease process, mapping intervention, and evaluating the outcome. One of the processes or pathways to evidence-based synthesis in medicine and public health remains meta-analysis or applied meta-analysis in the context of QES. *H. pylori*, during the past three decades, has been involved in gastritis and peptic ulcers, implying the inflammatory process resulting in abdominal bleeding. With an increased understanding of the role of inflammation in malignant neoplasm, *H. pylori* was implicated in gastric carcinoma; however, the causal mechanism, implying causal inference, of *H. pylori* and gastric carcinoma remain to be established. The current study assessed published literature in the process of generating evidence as to whether *H. pylori* is causative as a carcinogen in gastric carcinoma. We hypothesized that this pathogen transformed the pre-oncogene into an oncogene, as well as inactivated the tumor suppressor gene, therefore transforming the normal cells into abnormal proliferating cells in the gastric mucosa, resulting in gastric carcinoma. With the many factors involved in cancer development, there was a need to examine physical location or the environment to identify the contributory effect of *H. pylori* in gastric cancer development.

Table 1 illustrates the study characteristics of the sample with *H. pylori*, as well as controls for gastric carcinoma development in Eastern Asia, specifically South Korea. In this sample population, the variables studied sample size, as well as exposure size, and are illustrated in this table. In addition, the authors and the year of publication, along with the respective reference, indicate the study title, implying the outcome, exposure, as well as design. The study variables describe the sociodemographics, social determinants, as well as the laboratory techniques and the specimen utilized in the *H. pylori* detection as an exposure function of gastric carcinoma. In addition, other variables studied included lifestyles, such as smoking and alcohol consumption. Because of the multiple factors involved in cancer development, this table examined studies that control for confoundings and identified bias. The main confoundings in these studies included diet, family history of *H. pylori* and gastric cancer, and body mass index (BMI) as a marker of obesity. Table 2 illustrates the study characteristics, which are identical with Table 1, except for the geographic locale, reflecting Spain in Western Europe.

**Figure 2:**
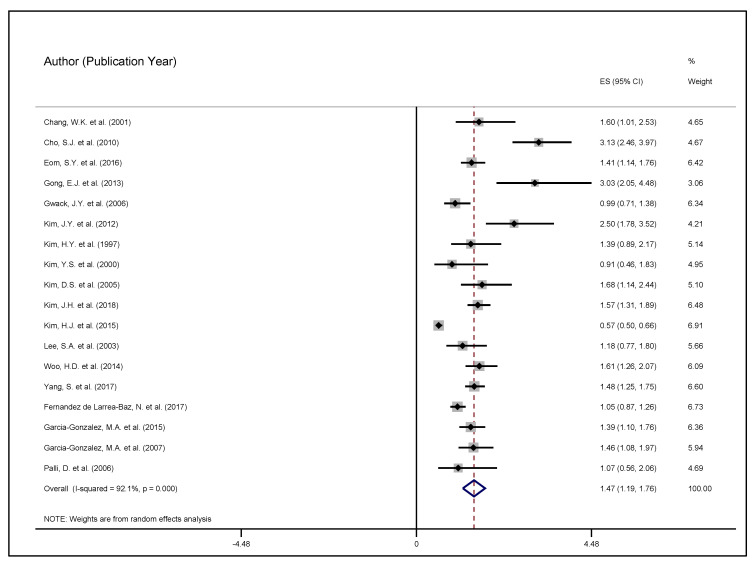
Forest plot of the correlation between *H. pylori* and gastric carcinoma. The diamond illustrates the common effect size (CES), while the error bars between the dots and the individual effect sizes represent the 95% lower and upper confidence intervals, respectively.

Figure 2 illustrates the summary or the pooled estimates in the Forest plot that characterizes the correlation between *H. pylori* as a carcinogen in gastric cancer development. There was a significant correlation between *H. pylori* and gastric cancer development in the overall sample of studies that constituted this applied meta-analysis, termed QES. Compared to the controls, the cases were 47% more likely to be exposed to *H. pylori* as a potential carcinogen in gastric cancer development, CES = 1.47, 95% CI, 1.19–1.76. The heterogeneity test, implying the variance between the CES and the individual effect sizes, was observed. There was a 92.1% significant heterogeneity, suggesting a substantial variability between the point-estimates in the individual studies compared to the summary estimates, χ^2^ (17) = 216.54, *p* < 0.01.

**Figure 3:**
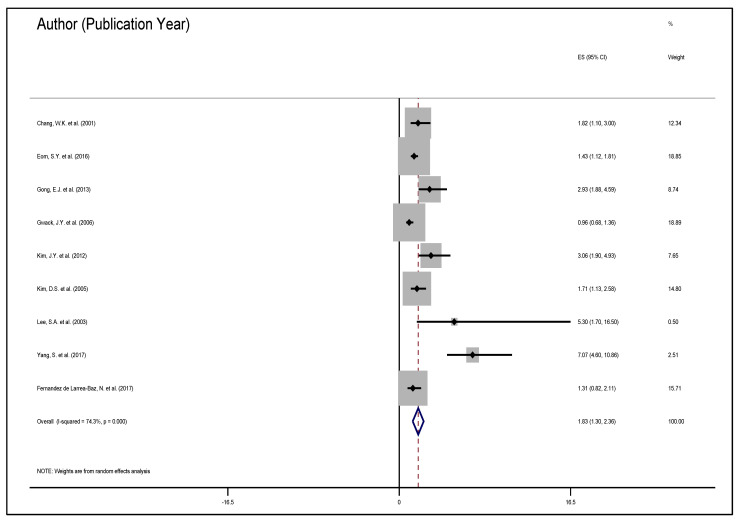
The correlation between *H. pylori* and gastric carcinoma adjusted for confoundings in a multivariable or multilevel analysis. The diamond illustrates the common effect sizes (CES) as the parameter estimate in the summary of the individual studies that constituted the CES. The dots represent the individual effect sizes, while the right and the left bars illustrate the 95% upper and lower limits, respectively.

Figure 3 represents the adjusted correlation between *H. pylori* and gastric carcinoma development. Since *H. pylori* per-se is not the only carcinogen in gastric malignancy, studies assessed and controlled for confoundings, implying the adjusted correlation between *H. pylori* and gastric carcinoma. With these studies, the summary or CES was observed to be 1.83, 95% CI 1.30–2.36. There was substantial heterogeneity (74.3%)—implying the variance between the CES and the individual effect sizes, χ^2^ (8) = 31.13, *p* < 0.001.

**Figure 4:**
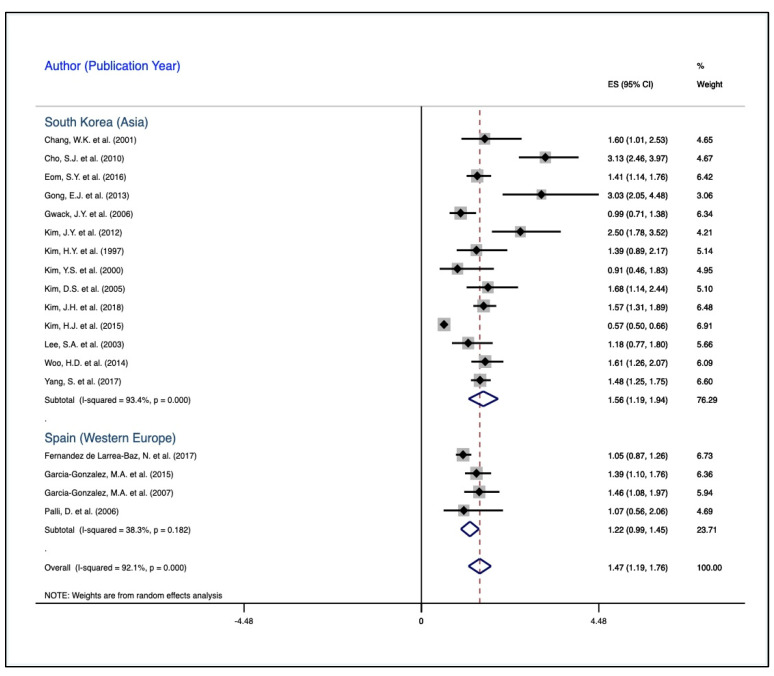
Meta-regression (subpopulation) correlation between *H. pylori* and gastric carcinoma. The subpopulations are South Korea (Eastern Asia) and Spain (Western Europe). The diamonds illustrate the common effect sizes (CES) for South Korea, Spain, and the overall population. The dots indicate the individual effect size, while the error bars represent the lower and the upper confidence limits as the measure of the precision of the individual effect sizes.

Figure 4 demonstrates the subpopulation correlation, termed meta-regression, between *H. pylori* and gastric carcinoma. Regardless of subpopulation, the forest plots illustrate a significant direct correlation between exposure to *H. pylori* and the development of gastric carcinoma. With respect to South Korea, compared to controls, the cases were 56% more likely to be exposed to *H. pylori*, CES = 1.56, 95% CI, 1.19–1.94. There was substantial heterogeneity, 93.4%, implying the variabilities between the individual effect sizes and the CES, χ^2^ (13) = 197.43, *p* < 0.001. Regarding Spain (Western Europe), compared to the controls, the cases were 23% more likely to be exposed to *H. pylori*, CES = 1.23, 95% CI, 1.00–1.45. There was an insignificant heterogeneity, implying a variability between the individual effect sizes and the CES, χ^2^ (3) = 4.86, *p* = 0.18. The above results are also displayed in Table 3.

The overall correlation between *H. pylori* and gastric carcinoma indicated a CES heterogeneity, implying that the effect size is not equal to 0, resulting in the rejection of the null hypothesis of homogeneity, that the effect size is equivalent to 0, z-statistic = 3.98, *p* < 0.001. The observed heterogeneity indicates a significant role of *H. pylori* in gastric carcinoma development. Similarly, there was an observed heterogeneity of the CES with respect to the studies that control for the confoundings in the correlation between *H. pylori* and gastric carcinoma, z = 6.77, *p* < 0.001, indicative of the significant contributory effect of *H. pylori* as a carcinogen in gastric mucosa after controlling for the potential confoundings, namely, diet, smoking, alcohol consumption, and BMI (Table 1 and Table 2). Regarding South Korean cases, the null hypothesis was rejected, with z = 8.23, *p* < 0.05. Similarly, the study in Spain observed a z-statistic of 10.45, *p* < 0.001, reflecting the rejection of the null hypothesis that the CES is not equal to zero.

## 4. Discussion

*H. pylori* has been implicated in gastritis and peptic ulceration during the past three decades. However, its implication in gastric carcinoma has not been well-established, suggesting the need for evidence-based data in understanding the exposure function of *H. pylori*, a gram-negative bacterial pathogen, in the development of gastric carcinoma. The current study was proposed to examine the evidence as to whether exposure to *H. pylori* increases the risk of gastric carcinoma. This study examined the null hypothesis using a quantitative evidence synthesis (QES), which is an applied meta-analysis that there is no association or correlation between *H. pylori* and gastric carcinoma development. The assessment of this nexus implied the application of a random effect meta-analysis in assessing the CES, heterogeneity between the CES, and the individual studies in the QES, as well as the z-statistics in assessing whether the CES equals zero. There are a few relevant findings based on this evidence from published literature, implying the study of studies and analysis of analyses. First, *H. pylori* is associated with increased gastric carcinoma. Secondly, a meta-regression based on geographic variation, observed as increased risk of gastric carcinoma with exposure function of *H. pylori*. Thirdly, regardless of controlling for confoundings, gastric carcinoma risk was associated with *H. pylori*.

We have illustrated in the studies utilized for the evidence with respect to scientific evidence and the role of *H. pylori* and gastric carcinoma. The synthesized data in this study implicate *H. pylori* in gastric carcinoma development. The observed evidence is also supported elsewhere [58,59]. The current QES observed an increased risk of *H. pylori* in several studies. This risk increased whether or not the individual studies adjusted for the confoundings in the correlation between *H. pylori* and gastric carcinoma.

Bacterial pathogens have been observed as pro-oncogenes, implying the ability to transform normal cells to abnormally proliferative cells characterized by poorly differentiated or undifferentiated cellular maturation. *H. pylori*, as a bacterial pathogen implicated in gastritis and peptic ulceration, due to the ability of this microorganism to infect the gastric mucosa, results in irritation and inflammatory response after infection. With the chronic inflammatory response involving the elaboration of inflammatory mediators, there is a tendency of impairment in the negative feedback mechanism that may involve DNA damage. The damage to DNA in this setting requires a repair mechanism, which, unfortunately, is not available. *H. pylori*, in effect, has a tendency of transforming the pro-oncogene to oncogene, as well as its inhibition of the activation of the tumor suppressor gene, namely, *p53*. The overall effect of this carcinogenic involvement in the gastric mucosa results in impaired apoptosis. Consequently, the implication of *H. pylori* in gastric carcinoma signals its invasion of the epithelial cells, resulting in epithelial proliferation, as well as apoptosis inactivation, which enables abnormal cellular proliferation of the gastric cells and subsequent gastric carcinoma.

We have also demonstrated that regardless of whether the individual studies that constituted this evidence-based synthesis assessed for the confoundings in the correlation between *H. pylori* and gastric carcinoma. The confoundings factors in gastric carcinoma development include the family history of gastric carcinoma, gastritis, peptic ulceration, smoking, diet, as well as other environmental carcinogens. The studies included in this QES control for diet, family history, and smoking, observing increased exposure to *H. pylori* among the cases compared to the control. The observed non-differential with respect to the risk of gastric carcinoma with the exposure function of *H. pylori* indicates evidence that this pathogenic microbe tends to invade the gastric mucosa and altering the cellular functionality to become neoplastic.

This study has also implicated that regardless of geographic variation, *H. pylori* associated with an increased risk of gastric carcinoma. The individual studies that comprised this QES involved two geographic locations, namely, South Korea (Eastern Asia) and Spain (Western Europe). While the geographic environment was utilized in this meta-regression, the implication of environment involves not only the physical environment, but the cultural, social, psychosocial, and chemical environment. Dietary intake varies across these two populations, as well as the healthcare delivery systems with respect to the diagnosis, detection, and management of *H. pylori*. In addition, the prevalence of *H. pylori* varies across these two populations. However, among South Koreans, there is a higher risk of *H. pylori* associated with gastric carcinoma compared to the Western Europeans (Spaniards). The question then remains, besides *H. pylori*, what are other potential carcinogens in gastric carcinoma in these two geographic locations? If the risk factors for gastric carcinoma vary by location, what are the major risks and predisposing factors in these two locations? Understanding these variabilities may facilitate intervention mapping in understanding the risk and the application of this knowledge in risk reduction.

Furthermore, since several epigenomic lesions are involved in malignancies, is it possible that epigenomic modulation involving *H. pylori* may inhibit the transcription factor, resulting in inverse gene expression and the subsequent abnormal protein synthesis and cellular dysfunctionality? Epigenomic mechanistic processes involve the interaction between the gene and the environment, implying the mechanistic process within the enhancer/promoter region of the gene characterized by the CpG islands, where the cytosine binds with the methyl group (-CH_3_), resulting in methyl-cytosines that inhibit the transcription factor and prevents the messenger RNA (mRNA) from translate the transcriptomes into amino acid codons, resulting in impaired protein synthesis and subsequent abnormal cellular proliferation. However, it is unclear of the contributory role of the DNA methyltransferase in the transfer of the -CH_3_ radical to the CpG region of the gene. Additionally, it is unclear whether or not the RNA polymerase is not enhancing the mRNA in the process of transcription.

Many malignancies are driven by mutation, implying that genetic mutations alter cellular function and can lead to abnormal cellular proliferation. Recently, however, epigenomic modulation, implying the gene and environment interaction, has been implicated in several malignancies. Understanding how genes interact with endogenous, exogenous, physical, chemical, social, psychosocial, and psychological environments allows for understanding how epigenomic genomic modulation results in dysfunctional cell division, differentiation, and maturation. While the genetics of *H. pylori* have been implicated in gastric malignancy, the interaction between the host gene and *H. pylori* may result in the DNA methylation of the host, namely, patients, resulting in hypermethylation of the CpG regions of the human genome, leading to the inhibition of the transcription factors (transcriptome), resulting in impaired gene expression, compromised protein synthesis, and abnormal cellular functionality. In effect, the recommendation of studies in this direction upon which the specific risk of being characterized with respect to the DNA methylation may facilitate induction therapy before the standard of care, such as monoclonal antibodies, surgery, radiation, or chemotherapy required in gastric carcinoma management.

Microbiome diversity, implying the trillions of microorganisms within the gastric intestinal tract (gut microbiome), has been implicated in obesity, type II diabetes, and cardiovascular disorders. The implication of microbiome diversity results from energy source and utilization, and when reflecting obesity, as well as type II diabetes. Specifically, the microbiome diversity reflects how the normal flora protects the human host from opportunistic infections, resulting in disease reduction and improved gastric intestinal activities. *H. pylori*, given the microbiome environment, is a pathogenic microbe classified by the immune system as antigenic. As the microbial diversity is received, there is an increased tendency for immunogenicity, resulting in the antibody production and the subsequent binding of the antibodies to the epitope of *H. pylori*, resulting in an immune-complex (IC) formation and the subsequent clearance of the IC by the complementary system activation via VRC3 (VR complement 3 (C3)), the critical component of the complementary system. The enhanced immunogenicity, given microbiome diversity, signals the immune system recognition of the *H. pylori*-induced cellular changes within the host and the elaboration of the tumor-specific anti-gene, resulting in the activation of the tumor suppressor gene and improved apoptosis. In addition, *H. pylori*, as a potential carcinogen, reflects its ability in transforming the pro-oncogene to the oncogene. While immunologic surveillance of malignancies, in general, requires the tumor-specific antigen recognition by the immune cells, *H. pylori* may reflect the loss of this surveillance by the immune cells, which reflects apoptosis inactivation, implying decreased activities of the tumor suppressor genes [60,61].

In general, microbiome diversity, comparing two populations such as Spain and South Korea, may play a role in gastric carcinoma development associated with *H. pylori*. In effect, the more diversified a population is in respect to microbiomes, the lower the risk of *H. pylori* resulting in gastric carcinoma. However, because gastric carcinoma risk factors do not occur in isolation, attempts to understand the multiple facets, with respect to the risk of gastric carcinoma, remains a pathway to gastric carcinoma reduction, prevention, as well as decreased mortality. While there are no definitive data on the implication of carcinogenic anti-microbial agents in cancer development, there are substantial data on the inhibition of the microbiomes and microbiome diversity reduction through the substantial prescription of anti-microbial agents provided to patients. Therefore, there is an increased probability of malignant neoplasms following resistant broad spectrum and combined anti-microbial agent therapy in gastric carcinoma development. This QES suggests population-based studies on the implication of anti-microbial agents in gastric carcinoma.

Evidence-based data, such as the applied meta-analysis termed the QES, allows for the generation of scientific statements, clinical and public health opinion. The current study has some strengths, despite the limitations. As an evidence-based study, the analysis of analyses for a common or pooled estimate indicates quantitative evidence discovery. Despite this strength, in the process of intervention mapping, there are some limitations. First, the QES, which involves the study of studies, is a retrospective design, implying the potential for observational, informational, selection, and misclassification biases. We examined the individual studies that constituted this meta-analysis for evidence of differential misclassification bias. However, it is highly unlikely that the evidence in these findings, where *H. pylori* is implicated in gastric carcinoma, results from these biases.

## 5. Conclusions

In summary, this quantitative evidence synthesis, as an applied meta-analysis, indicates a direct correlation between *H. pylori* and gastric cancer development, implying increased exposure to *H. pylori* among cases compared to the controls, which is highly suggestive of the excess risk of gastric carcinoma following exposure to *H. pylori*. The observed findings require intervention mapping in reducing or minimalizing infection from *H. pylori* through early diagnosis, anti-microbial therapy, as well as H2 blockers. With respect to the geographic variation, this QES encourages the World Health Organization (WHO) to allocate funding in addressing the disproportionate benefit in addressing *H. pylori* infection in South Korea relative to Spain.

## Figures and Tables

**Table 1:** Causal association of *Helicobacter pylori* and gastric cancer in South Korean studies.

Author & Date	Sample Population	Sample Size (Exposure)	Variables Studied	Measure of Effect	Results
Chang et al., 2001 [42]	136 GC, 13 controls	126 exposed, 146 unexposed	Age, sex, marital status, occupation, education, salary, *H. pylori* serology	OR 1.82, (1.10–3.00)	+ effect
Cho et al., 2010 [43]	2819 GC, 564 controls	2763 exposed, 618 unexposed	Age, sex, smoking, drinking, family history, living conditions, education, SES, family structure	OR 3.13, (2.46–3.97)	+ effect
Eom et al., 2016 [44]	846 GC, 846 controls	1252 exposed, 440 unexposed	Age, sex, smoking, drinking, education, BMI, *H. pylori* infection, cagA	OR 1.43, (1.12–1.81)	+ effect
Gong et al., 2013 [45]	327 GC, 327 controls	448 exposed, 169 unexposed	Age, sex, BMI, *H. pylori* serology, family history, smoking, drinking	OR 2.93, (1.88–4.59)	+ effect
Gwack et al., 2006 [46]	100 GC, 400 controls	449 exposed, 51 unexposed	Age, sex, smoking, drinking, education, diagnosis, *H. pylori* serology, cagA and vacA	OR 0.96, (0.68–1.36)	N/A
Kim et al., 2012 [47]	829 GC, 270 controls	917 exposed, 180 unexposed	Age, sex, smoking, drinking, family history, BMI, *H. pylori* serology, eradication efforts, past diagnoses	GCC, OR 2.50, (1.78–3.52) GNCC, OR 2.10, (0.98–4.48)	+ effect
Kim et al., 1997 [48]	160 GC, 160 controls	179 exposed, 141 unexposed	Age, sex, *H. pylori* serology	OR 1.39, (0.89–2.17)	+ effect
Kim et al., 2000 [49]	287 GC, 33 controls	142 exposed, 178 unexposed	Age, sex, urbanity, SES, education, BMI, *H. pylori* serology, smoking, drinking	43.9% GC group, 48.5% non-GC group	N/A
Kim et al., 2005 [50]	295 GC, 295 controls	N/A	Age, sex, SES, education, BMI, *H. pylori* serology, smoking, drinking, family history	OR 1.68, (1.14–2.44)	+ effect
Kim et al., 2018 [51]	415 GC, 830 controls	868 exposed, 353 unexposed	Age, sex, BMI, *H. pylori* serology, family history, smoking, drinking, exercise, education, marital status, occupation, salary, diagnoses	92.1% GC 58.6% non-GC	+ effect
Kim et al., 2015 [52]	998 GC, 1288 controls	1817 exposed, 469 unexposed	Age, sex, BMI, smoking, drinking, family history, *H. pylori* serology, diagnosis	M: 86.2% GC M: 77.1% non-GC W: 87.2% GC W: 71.1% non-GC	+ effect
Woo et al., 2014 [53]	334 GC, 334 controls	457 exposed, 211 unexposed	Age, sex, BMI, *H. pylori* serology, family history, smoking, drinking, exercise, education, marital status, occupation	84.4% GC 52.4% non-GC	+ effect
Yang et al., 2017 [54]	450 GC, 1050 controls	1051 exposed, 449 unexposed	Age, ALDH2 genotype, drinking, smoking, education, salary, diet, *H. pylori* serology, family history	OR 7.07, (4.60–10.86)	+ effect

**Table 2:** Causal association of *H. pylori* and gastric cancer in Spanish studies.

Author & Date	Sample Population	Sample Size (Expose)	Variables Studied	Measure of Effect	Results
Fernandez de Larrea-Baz et al., 2017 [55]	264 GC, 2071 controls	2017 exposed, 259 unexposed	Age, sex, race, education, SES, BMI, smoking, family history, diagnosis	OR 1.31, (0.82–2.11)	+ effect
Garcia-Gonzalez et al., 2015 [56]	603 GC, 675 controls	794 exposed, 484 unexposed	Age, sex, smoking, family history, cagA vagA	OR 1.39, (1.10–1.76)	+ effect
Garcia-Gonzalez et al., 2007 [57]	404 GC, 404 controls	506 exposed, 302 unexposed	Age, sex, smoking, family history, cagA vagA	OR 1.46, (1.08–1.97)	+ effect

Abbreviations for Table 1 and Table 2: Gastric carcinoma (GC); gastric non-cardia carcinoma (GNCC); odds ratio (OR); body mass index (BMI); aldehyde dehydrogenase (ALDH2); socioeconomic status (SES); positive (+); men (M); women (W).

**Table 3:** Unadjusted and pooled point-estimates from studies in South Korea and Spain with regards to *H. pylori* infection and gastric carcinoma development.

Author and Publishing Year	ES (OR)	95% CI		Weight
South Korea (Asia)				
Chang, W.K. et al. (2001)	1.60	1.01	2.53	4.65
Cho, S.J. et al. (2010)	3.13	2.46	3.97	4.67
Eom, S.Y. et al. (2016)	1.41	1.14	1.76	6.42
Gong, E.J. et al. (2013)	3.03	2.05	4.48	3.06
Gwack, J.Y. et al. (2006)	0.99	0.71	1.38	6.34
Kim, J.Y. et al. (2012)	2.50	1.78	3.52	4.21
Kim, H.Y. et al. (1997)	1.39	0.89	2.17	5.14
Kim, Y.S. et al. (2000)	0.91	0.46	1.83	4.95
Kim, D.S. et al. (2005)	1.68	1.14	2.44	5.10
Kim, J.H. et al. (2018)	1.57	1.31	1.89	6.48
Kim, H.J. et al. (2015)	0.57	0.50	0.66	6.91
Lee, S.A. et al. (2003)	1.18	0.77	1.80	5.66
Woo, H.D. et al. (2014)	1.61	1.26	2.07	6.09
Yang, S. et al. (2017)	1.48	1.25	1.75	6.60
Sub-Total D + L Pooled ES	1.56	1.19	1.94	76.29
Spain (Western Europe)				
Fernandez de Larrea-Baz, N. et al. (2017)	1.05	0.87	1.26	6.73
Garcia-Gonzalez, M.A. et al. (2015)	1.39	1.10	1.76	6.36
Garcia-Gonzalez, M.A. et al. (2007)	1.46	1.08	1.97	5.94
Palli, D. et al. (2006)	1.07	0.56	2.06	4.69
Sub-Total D + L Pooled ES	1.23	1.00	1.45	23.71
Overall D + L Pooled ES	1.47	1.19	1.76	100.00

## Data Availability

The data presented in this study are available in the referenced articles [42,43,44,45,46,47,48,49,50,51,52,53,54,55,56,57].
